# A Multi-Modality Intervention Improves Obesity Bias among Medical Students

**DOI:** 10.3390/medicines11020004

**Published:** 2024-01-28

**Authors:** Stephanie Trofymenko, Randa Kutob, Amit Algotar

**Affiliations:** 1 Department of Biomedical Sciences, Noorda College of Osteopathic Medicine, 2162 S 180 East, Provo, UT 84606, USA; 2 Department of Family & Community Medicine, College of Medicine, University of Arizona, 655N Alvernon Way, Suite 228, Tucson, AZ 85711, USA; rkutob@arizona.edu (R.K.); algotar@arizona.edu (A.A.)

**Keywords:** medical education, obesity bias, teaching, education, obesity

## Abstract

**Background**: Obesity is linked to chronic diseases in adults and children. Its prevalence continues to grow in the United States, necessitating the need for healthcare provider training and presenting an opportunity for the education of future medical providers. Despite this need, effectively implementing obesity education into medical school curricula has been challenging. Anti-obesity bias amongst healthcare providers and trainees represents a significant obstacle to the care of patients with obesity. Obesity bias may affect up to 1/3 of medical students. **Methods:** This study describes the development and preliminary testing of a brief, 2.5 h multi-modality teaching intervention consisting of online, interactive, and independent learning modules for first-year medical students and a patient panel focused on obesity, obesity bias, and motivational interviewing. The participants took Crandall’s anti-fat attitude (AFA) questionnaire before and after an online independent learning module on motivational interviewing and obesity bias. The AFA consists of three subscales (“dislike”, “fear of fat”, and “willpower”). Individual responses were measured using a nine-point Likert-type response format (0 = very strongly disagree; 9 = very strongly agree). An average composite score was calculated for each subscale. **Results:** Data were analyzed from 103 first-year medical students enrolled at a college of medicine in the southwestern United States in 2022. The AFA mean composite scores decreased significantly, indicating a decrease in explicit anti-obesity attitude bias after completing the online module. This decrease was present in all three domains of fear (4.63 vs. 3.72, *p* < 0.001), dislike (1.25 vs. 0.88, *p* < 0.001) and willpower (3.23 vs. 2.31, *p* < 0.001). **Conclusions:** Relatively brief educational interventions can positively impact students’ anti-obesity attitudes.

## 1. Introduction

Obesity, as defined by a body mass index (BMI) of ≥30 kg/m^2^, affects 41.9% of the adult US population [1]; a prevalence rate that has been steadily increasing from 30.5% in 1999 [2]. Obesity rates in children are also concerning. Nearly 20% of U.S. children and adolescents between the ages of two and nineteen were reported to be obese in 2017 [2]. Obesity is linked to many chronic diseases in adults and children, making addressing obesity of dire importance, as it poses a significant threat to public health [2]. In order to properly address this growing epidemic, there is an opportunity for the education of future medical providers.

Obesity education is often not prioritized in medical school curricula. In a study that surveyed forty medical schools, only an average of 10 h of dedicated obesity education was available to the students. One-third of the schools surveyed reported no current obesity education and no plans to implement this topic. Half of the schools reported that expanding obesity education was not a priority [3]. These findings stand in stark contrast to the reality of obesity in the United States. Obesity exists as part of a continuum that is often preceded by social determinants of health and results in health disparities with downstream effects in a myriad of chronic diseases [4]. Knowledge of obesity and obesity treatment is vital not only for primary care but also for medical specialists, as well [4]. The Association of American Medical College (AAMC) acknowledged the importance of weight management in 2007, and its guiding principles recommend that this topic be emphasized in medical school curricula [5].

Effectively implementing obesity education into medical school curricula has been challenging, due in part to the nature of the disease of obesity. Some of the barriers that exist in training students to address patients with obesity appropriately and effectively include a lack of knowledge by curriculum developers regarding how best to integrate these topics into the medical curriculum. These topics include effective interventions [6], a lack of confidence in managing obesity [7], a perceived lack of motivation of patients to lose weight from physicians [7], and the negative bias towards people with obesity [8,9,10,11,12]. Eliminating or mitigating these biases from students through different educational models in medical school is crucial, with the potential to have a positive effect on future patient–provider relationships as well as patient outcomes. 

Understanding bias in medical students is an important step in addressing obesity education and improving patient outcomes [13]. A study by Miller et al. of more than 300 third-year medical students reported that over one-third of the medical students had significant implicit anti-obesity bias. Only a few were aware of that bias [14]. A different study by Essel et al. of second-year medical students demonstrated that most of them had a negative implicit bias towards individuals with obesity [15]. Upon self-reflection, these students struggled to accept their weight attitudes implicit test results, indicating that many students may not be aware of their biases [15]. Many medical students feel uncomfortable discussing obesity [16], and may hold negative attitudes towards people with obesity, which could interfere with their ability to provide effective treatment. A study of medical professionals reported that implicit anti-obesity attitudes have decreased in the last decade, although explicit anti-obesity attitudes have increased. This same study compared the measured explicit anti-obesity bias in 2001 and 2013 of attendees at the Obesity Week conferences [17]. Another report concluded that even professionals who work in a clinical or research capacity related to treating obesity have a strong implicit bias [18], indicating that there is a powerful stigma against individuals living with obesity. In an interventional study of several cohorts of first-year medical students, 70% of the students held a thin preference. In this study, the students were surveyed to assess their obesity attitudes before and after an imbedded course focused on improving their attitudes through an ethics session. During the educational ethics session, the students discussed topics related to their beliefs about the causes of obesity, as well as their personal struggles with weight. In this same study, 74% of the students surveyed believed that obesity came from ignorance, and nearly 30% of the students thought people with obesity were lazy [19]. In a national sample of first-year medical students, most students demonstrated having explicit and implicit obesity bias. They reported that explicit attitudes were more negative towards people with obesity than to other marginalized groups, such as racial minorities, gays, lesbians, and poor people [20].

Amongst clinicians, although it is known that negative obesity bias exists, only a few effective interventions have been reported, representing a significant gap in the medical education literature [16]. Only a few studies were found to have successfully used a variety of interventional teaching methods, including the use of standardized patients with obesity [21], ethics combined with popular media [19,22], and nontraditional teaching methods such as theatre [23] to increase medical students’ skills, knowledge, and attitudes regarding obesity in patients [21,22,24]. One study found positive outcomes in third-year medical students by pairing them with patients undergoing bariatric surgery. This study measured their attitude toward obesity and provided faculty mentorship throughout the year that the students were paired with patients experiencing obesity who were undergoing bariatric surgery [25]. These techniques have the potential to decrease obesity bias and increase medical students’ skills and understanding in addressing patients with obesity. Another study reported that anti-obesity prejudice can be reduced, depending on the information provided about the disease process of obesity, with regards to controllable vs. uncontrollable factors [20]. The medical students included in these studies were more likely to believe that obesity is caused by controllable factors, such as diet and inactivity, instead of biological factors [26]. Attributing obesity to external factors was correlated with a greater ability to counsel patients with obesity [26]. Phelan et al. reported that medical student implicit bias decreased, as reported using the weight implicit association test, while explicit bias increased, which was measured with a validated feeling thermometer towards individuals with obesity during their four years of study [27]. In their study, they reported that implicit bias decreased significantly as the students participated in more positive interactions with patients with obesity [27], demonstrating the need for students to be better prepared to address patients with obesity. 

The authors created a brief educational intervention consisting of a patient panel and an online module on obesity prevalence, bias, and counseling patients with obesity utilizing motivational interviewing and the 5 As model. The participants (first year medical students) completed a validated self-report obesity bias survey immediately before and after completion of the online module. The hypothesis was that this brief intervention, specifically the online module, could decrease self-reported obesity bias, as measured by a validated survey instrument. 

## 2. Materials and Methods

### 2.1. Online Course Development and Content

An interactive web course using a case-based learning model was developed by the authors (Algotar and Kutob), who are both primary care and obesity medicine physicians. Both authors are diplomates of the American Board of Obesity Medicine. The learning objectives were to:

1. Incorporate knowledge of prevalence of obesity in the United States into practice.
2. List two strategies to mitigate weight bias in clinical settings.
3. Utilize four motivational interviewing techniques in clinical settings.
4. Identify the steps of the 5As behavior change model and apply these to lifestyle counseling in a clinical scenario.

The course introduced a fictional patient, Maria Chavez, a 42-year-old woman with knee pain and obesity, and described her interactions at a clinic visit with medical personnel starting at intake and continuing throughout the visit with a physician. Through interactive questions with answer feedback, the learners were introduced to information on U.S. obesity trends, obesity bias in medical settings, and strategies to mitigate this bias. At Ms. Chavez’s second visit, the learner became the “physician” in the scenario and was introduced to motivational interviewing (MI). Motivational interviewing, as defined by Miller and Rollnick, is a “a collaborative conversation style for strengthening a person’s own motivation and commitment to change” [28]. MI was originally developed in the context of helping patients with substance use disorder, but has subsequently been applied to a variety of health conditions and behaviors including smoking cessation, diabetes management, obesity treatment, vaccine hesitancy, and others [29,30,31,32,33,34]. The course provided information on the key MI concepts of partnership, acceptance, compassion, and evocation [28]. The students were introduced to motivational interviewing and were taught four steps of motivational interviewing, which included engagement, focusing, evoking, and planning. Engagement was introduced as being the key step that sets the stage for the patient–physician interaction, involving asking open-ended questions, affirming positive aspects of patient statements, reflection, and summarizing information. Focusing was introduced as involving a collaboration with the patient. Evoking was introduced as involving a process of exploring ambivalence. The course allowed the students to guide the patient in the scenario through evoking “change talk” (language that a patient or client might use that is an argument for change) and understanding the differences between “change talk” and “sustain talk” (language that is directed to maintain the status quo). Planning was introduced as the steps that are involved in helping patients develop a plan of action based on their goals. The students were introduced to five steps of planning, including confirming the goals and sub-goals along the way, itemizing options available or ones that have been discussed, eliciting the patient’s views on the best way forward, summarizing the plan and discussing the commitment to the plan, and troubleshooting the plan. The learners were then guided through the steps of the MI process including engaging, focusing, evoking, and planning [28], with opportunities to apply these interactively during Ms. Chavez’s visit. Finally, the learners were introduced to the 5 As behavior change model originally developed by the U.S. Department of Health and Human Services in the context of smoking cessation [35]. The course included nine embedded questions throughout the patient scenario to guide the students’ understanding of implementing the 5As as well as correctly using MI techniques. 

The course took approximately 1 h to complete and was hosted on the university’s continuing medical education (CME) website (https://www.vlh.com/, accessed on 28 March 2022). The course was a mandatory independent learning module for the first-year medical students and was also available to providers both inside and outside the university for CME and maintenance of certification credit. The students were given one hour of curricular time to complete the module.

### 2.2. Patient Panel

On the same day as the required independent learning module, the students attended a required 1.5 h long session, which included a panel of patients undergoing current or prior treatment for obesity. Attendance for the patient panel was mandatory; however, answering the survey and participating in the research was voluntary. Due to the COVID-19 pandemic, the session was conducted virtually via Zoom^®^. The students received a brief introduction to the session by the online course faculty, which included a brief review of the online course described above. Following this introduction, the panelists were asked to comment on how they felt the diagnosis of obesity affected their healthcare experience. The panel consisted of three volunteers that were drawn from the authors’ clinical practices. The following questions were asked to the panelists to begin the discussion:

1. Do you feel your weight has been a factor in your interactions with the healthcare system and the kind of care that you have received?
2. Has weight affected your interactions outside of the healthcare system?

During the discussion, the students attending the panel had the opportunity to ask questions. The closing of the discussion was marked by the panelists sharing how they believed their negative experiences with the healthcare system could have been improved. 

### 2.3. Study Population and Experimental Design

Data were collected from 110 first-year medical students enrolled at a college of medicine in the Southwestern U.S. in 2022. Seven students were excluded from the study because they did not complete a post-test, leaving the study sample as *N* = 103. This project was reviewed by the university’s Institutional Review Board and deemed not to be research. The data collected included answers to demographic questions including age; race/ethnicity; gender; knowledge of guidelines for the treatment of obesity, ranked from excellent to poor; and an estimated total number of hours in training they had in weight management counseling. 

### 2.4. The Anti-Fat Attitudes Questionnaire

The participants completed Crandall’s anti-fat attitude (AFA) questionnaire. This validated measure examines explicit attitudes towards people with obesity and obesity in general [34]. All the items were measured in a 9-point Likert-type response format (0 = very strongly disagree; 9 = very strongly agree). Higher scores indicated stronger anti-obesity attitudes [34]. A notification was included in the introduction to the survey regarding the use of the term “fat”. The notification alerted the students to the use of the term and that the term was retained to maintain the validity and reliability of the original survey instrument. The AFA consists of three subscales (“dislike”, “fear of fat”, and “willpower”) [36,37]. Table 1 illustrates the anti-fat attitudes questionnaire questions and subscales. 

For each subscale, an average composite score was calculated by adding the scores from the subscale and dividing them by the number of items in the subscale. The AFA was embedded into the online course and administered both immediately before and after the course content. An online informed consent statement was included prior to the AFA. The students were informed that answering the survey questions and completing the course was voluntary. The students received 1 h of curricular time before the patient panel to complete the online module. 

### 2.5. Statistical Analysis

This study’s primary hypothesis was that first-year medical students taking the module would report decreased obesity biases. The AFA individual domain (dislike, fear of fat, and willpower) mean scores were the primary study endpoints. Paired *t*-tests were used to compare changes in the domain scores for each group between the baseline and post-testing conditions. All the analyses were performed using IBM SPSS, Version 28.

## 3. Results

Most of the participants identified as female (53%). White/non-Hispanic (47%) and Hispanic (18%) were the most identified racial/ethnic backgrounds. In addition to demographic data, Table 2 displays information regarding the participants’ knowledge of obesity treatment guidelines and hours of prior training with regards to obesity. Most of the participants (88%) reported average, less than average, or poor knowledge of obesity treatment guidelines, and 63% had 1 h or less of prior training. 

The course faculty intended for the students to complete the online course module prior to the patient panel, and curricular time was made available to do this. However, based on the recorded time, most of the students completed the online module including the pre- and post-questionnaire after the patient panel. 

As shown in Table 3, the AFA mean composite domain scores decreased significantly, indicating a decrease in explicit anti-obesity attitude bias after completing the online module. This decrease was present in all three domains of fear (4.63 vs. 3.72) *p*-value < 0.001, dislike (1.25 vs. 0.88) *p*-value < 0.001, and willpower *p*-value < 0.001 (3.23 and 2.31). Cohen’s *d* ranged from 0.42 to 0.90, indicating a medium to large effect size, with the greatest increase in the Willpower domain.

## 4. Discussion

Using a multi-modality intervention, this study demonstrates improvements in medical students’ self-reported explicit bias towards patients with obesity, with an improvement in all three domains of the anti-fat attitudes questionnaire. The intervention included a panel of patients with obesity who shared their experiences in the health care system, followed by an online educational module which placed a strong emphasis on recognition of obesity bias in health care and motivational interviewing techniques to counsel patients. The course learning objectives were met through a scenario of a patient with obesity. The 5As technique strategies and motivational interviewing were assessed through embedded questions throughout the patient scenario. These components were implemented to provide the students with background and strategies such as motivational interviewing, which will help them to address patients with obesity. The key components of motivational interviewing were introduced to the students as being rooted in the thought that patients are the experts on themselves. The key aspects of motivational interviewing introduced to the students during the modules were partnership, acceptance, compassion, and evocation [28]. The course included a patient scenario with nine embedded questions with explanations to help assess student understanding throughout the module. The online course to mitigate explicit anti-obesity attitudes among medical students was demonstrated to be a promising method and efficient educational tool. 

Other studies have had similar positive results in decreasing obesity bias using educational interventions, such as implementing short intervention models among residents, as well as other approaches. In a study assessing pediatric residents’ obesity bias, positive results were reported upon implementing an obesity curriculum consisting of reading material, videos, and lectures [38]. Positive results from existing intervention studies suggest that they can be used as effective tools to improve student attitudes towards people with obesity [19,20,21,22,23]. Some interventional studies included data on first-year medical students using a modality of interventional techniques, including an ethics session, the use of a popular T.V. show [19], interacting with standardized patients with obesity, reading material [21], and implementing dramatic reading [23] to decrease measures of explicit anti-obesity bias. Although some studies have reported positive results when implementing interventional studies for other medical students, there are few studies that have focused directly on reducing negative attitudes, negative stereotypes, bias, and increasing knowledge of obesity and learning to effectively address patients with obesity. Many studies in the past have focused on other aspects of treatment related to patients with obesity, including technical skills, such as the ability of students to interpret waist circumference and assess total body composition, as well as knowledge of obesity and obesity management [24]. 

In a packed medical school curriculum, finding curricular time is an ongoing challenge. The current study demonstrates that even a brief intervention can yield positive results. Medical students are in a unique position and are the forefront of future medical providers. Properly training the next generation on how best to address patients with obesity could result in positive outcomes in patient health and patient–physician satisfaction. Incorporating these educational intervention methods into medical school curricula presents a significant opportunity to fill this educational gap. 

There is a strong need to focus more on teaching about obesity treatment and reducing bias. Although obesity rates have been continually increasing in the United States, studies have reported a decline in addressing weight during clinical visits [39,40,41,42]. Research suggests that physicians in training are not always able to recognize obesity, spending a relatively small amount of clinic time treating obesity and not being adequately educated regarding treatment options [16,43]. 

Additionally, multiple studies have shown significant negative obesity bias among students and even healthcare professionals. Patients with obesity are often treated differently than patients without obesity in healthcare settings [9,10,11], which may influence the quality of healthcare provided to patients with obesity. Patients with obesity are often targets of humor by attendings, residents, and students [44]. A survey of more than 600 physicians reported that physicians share society’s broader negative stereotypes about obesity and view it as a behavioral problem. A study reported that most physicians rated obesity treatment as less effective than therapies for 9 of 10 other chronic conditions [45]. Another survey sample of more than 2000 physicians revealed that doctors have significant implicit and explicit anti-obesity biases [12]. Physicians also establish less emotional rapport with patients who are overweight or obese [46], affecting patient outcomes. Positive outcomes in weight and overall health are associated with physician advice and counseling through motivational interviewing [47]. The online course dealt directly with obesity bias but also included an introduction to motivational interviewing in the context of obesity and the opportunity to practice this skill. It is possible that the motivational interviewing content contributed to the decrease in explicit bias as measured by the AFA by allowing the students a safe place to practice this skill as they progressed through an online scenario. 

The limitations to this study include a single group pre–post design without a control group and the possibility of social desirability bias affecting the students’ answers to the AFA. Also, although the course faculty intended for the students to take the online course prior to the patient panel, almost all of the students completed the course a few hours after the patient panel, even though they were given curricular time to do so before. This could have affected the AFA pre-module scores if the participants’ bias was already reduced by attendance at the patient panel, thus reducing the magnitude of pre-/post- changes on the AFA assuming higher (more explicit bias) scores had the AFA been administered prior to the patient panel. The fact that significant changes occurred between the pre-test administered immediately before the module and the post-test administered immediately after the module indicates that the module itself had an effect, which may have been even greater if not preceded by the patient panel. 

There are many promising ways in which medical schools can implement obesity educational tools that mitigate obesity bias and support lifestyle counseling techniques such as motivational interviewing. The results of this study indicate that even relatively brief educational interventions that take little curricular time can positively impact students’ anti-obesity attitudes. To advance patient and community care, it is vital to provide aspiring physicians with the necessary tools to effectively address their patients’ distinct needs. As obesity rates continue to rise in the United States, it is important for medical education to adapt to meet the needs of patients and communities more effectively.

## Figures and Tables

**Table 1:** The anti-fat attitudes questionnaire questions and subscales.

Dislike	Willpower	Fear of Fat
I really don’t like fat people much.	Fat people tend to be fat pretty much through their own fault.	I feel disgusted with myself when I gain weight.
I have a hard time taking fat people seriously.	Some people are fat because they have no willpower.	I worry about becoming fat.
Fat people make me feel somewhat uncomfortable.	People who weigh too much could lose at least part of their weight through a little exercise.	One of the worst things that could happen to me would be if I gained 25 pounds.
I don’t have many friends that are fat.		
I tend to think that people who are overweight are a little untrustworthy.		
Although some fat people are surely smart, in general, I think they tend not to be quite as bright as normal weight people.		
If I were an employer looking to hire, I might avoid hiring a fat person.		

**Table 2:** Study participants’ demographics/characteristics, N = 103.

	% (n)
Gender female/male	Female 53% (55)/Male 48% (48)
Mean age (years)	24.8
Race/ethnicity	
White, Non-Hispanic	47% (48)
Hispanic	18% (19)
Black, African American	8% (8)
Asian	12% (12)
American Indian or Alaska Native	5% (5)
Other Race/ethnicity	11% (11)
Knowledge of obesity treatment	
Excellent	5% (5)
Above Average	8% (8)
Average	40% (41)
Below Average	33% (34)
Poor	15% (15)
Prior Obesity Training	
Less than 1 h	63% (65)
1–3 h	20% (21)
4–6 h	10% (10)
7–10 h	2% (2)
10 or more hours	5% (5)

**Table 3:** Results of paired *t* tests for mean composite domain scores.

Domain	Pre (Mean)	SD	Post (Mean)	SD	*p*-Value	Cohen’s d	Cohen’s d Confidence Interval (Lower, Upper Limit)
Fear	4.63	2.34	3.72	2.60	<0.001	0.61	0.44; 0.86
Dislike	1.25	1.30	0.88	1.15	<0.001	0.42	0.22; 0.62
Willpower	3.23	1.90	2.31	1.87	<0.001	0.90	0.67; 1.12

## Data Availability

Data is contained within the article.
